# The Talk of a Dentist

**Published:** 1885-10

**Authors:** 


### The Talk of a Dentist.

HOW WORK ON THE TEETH HAS BEEN PERFECTED—ADVANCE OF SCIENCE.

"A ton of gold goes under ground nearly every year," said a prominent Philadelphia dentist, "buried in the teeth and plates of people who have at one time or another been in the dental chair. The repair and refurnishment of the teeth has got to be a profession of the highest skill and proficiency. High standing in the profession is repaid with the richest rewards. The establishment of the university department of dentistry has given a great impetus to the study. Scores of able and expert young men matriculate annually. They come from all parts of the world—South America, Cuba, Mexico, the continent and Japan. This city is foremost in dental operations and dental surgery. Some of the work turned out here is wonderfully perfect. Many men and women prefer false teeth to the natural ones, if the latter are the least bit defective, and few people have a perfect set of teeth.

"Instruments? Why, yes, the instrumentation of a first-class dentist is comprised in several large cases, like that," pointing to a series of handsome rosewood cases, and pulling out drawer after drawer, filled with delicate steel probes, chisels, borers, and forceps. "The manufacture of these is a great trade in itself. There is the dental engine, one of the greatest inventions in the profession, indispensable now, with its flexible screw. The electric mallet, another modern invention unknown to the old-fashioned tooth-carpenters, is used by nearly all dentists and requires a battery to run it. The robber dam or appliance placed over the tooth and mouth of a patient to prevent moisture and saliva reaching the part operated on, is the greatest of the modern discoveries. Anyone who has been in a dentist's chair under the old plan, which necessitated packing the mouth of the patient with napkins, and since, under the rubber dam, can see what infinite torture this scientific adaptation has relieved him from.

"Twenty thousand dollars a year? Yes, there are dental surgeons in this city who make that much by their profession. A clientage very often includes a whole family and the care of the teeth of each from infancy until adolescence and beyond. American dentists have the highest repute abroad—Dr. Evans, for instance, whose patients in Paris and elsewhere were Empresses, Kings, Queens and princes of the blood.

"Gold is the best material yet found for filling teeth. Silver and compositions of various kinds, being cheaper, are used, but the royal metal is the only one which ought to be used. The manufacture or gold foil or leaf for our business is immense, and hundreds of thousands of dollars worth are consumed every year.

"The teeth should be looked to often by a good dentist. Individual care early in life saves much dental work and expense. It used to be the idea that the deciduous teeth, as they were temporary affairs, needed no attention. They should be treated with greater attention than the second set. They are not filled now as much as formerly, but extracted when caries attack them. The biblical expression, 'skin of the teeth,' is true. There is a delicate enamel, resembling epidermis in its microscopic delicacy, and covering the teeth with a beautiful mosaic, which is susceptible of a perfect polish, which you may see glistening on the teeth of some young people and Africans. Acids go for this, and once broken in upon caries ensues. Good and bad teeth are hereditary, but early care and professional skill will do much with even a bad natural set of teeth. A Philadelphia father I know—client of mine—has in each of his children's rooms over the lavatory the following motto: 'Say your prayers; wash your face; comb your hair; brush your teeth.' It is a good one."—*Philadelphia Times.*

